# Vegetation C: N: P stoichiometry and ecosystem carbon storage under different grazing patterns on the Tibetan Plateau

**DOI:** 10.3389/fpls.2025.1651605

**Published:** 2025-11-26

**Authors:** Guoxing He, Xiaoni Liu, Yali Li, Tong Ji

**Affiliations:** 1 Key Laboratory of Grassland Ecosystem, Ministry of Education, Pratacultural College, Gansu Agricultural University, Lanzhou, Gansu, China; 2 Sino-U. S. Center for Grazing Land Ecosystem Sustainability, Lanzhou, Gansu, China

**Keywords:** alpine meadow, grazing patterns, plant functional group, vegetation stoichiometry, ecosystem C storage, Tibetan Plateau

## Abstract

**Introduction:**

Grazing is a significant driver of grassland ecosystems changes, but the relationship between plant functional groups’ carbon (C): nitrogen (N): phosphorus (P) stoichiometry ratios and ecosystem C storage under different grazing management patterns remains uncertain.

**Methods:**

This study investigated alpine meadows on the northeastern edge of the Tibetan Plateau, comparing four grazing patterns: banned grazing (BG), growing season rest-grazing (RG), traditional rest-grazing (TG), and continuous grazing (CG). We assessed the effects of these grazing patterns on plant functional group nutrient, C: N: P stoichiometry, and ecosystem C storage. The results provided valuable insights to support sustainable management strategies in alpine meadow ecosystems.

**Results and discussion:**

The results indicated that BG and RG enhanced nutrient enrichment, vegetation, soil, and ecosystem C storage. BG, RG, and TG increased aboveground C: N ratios but decreased C: P and N: P ratios. Grazing patterns indirectly influence ecosystem C storage by regulating plant stoichiometry and biomass allocation among functional groups. AGB of grasses is significantly positively correlated with ecosystem C storage (*R*² = 0.96), serving as the key driver of changes in C storage. In conclusion, maintaining biomass balance among functional groups and the C: N: P stoichiometry of plants is vital for preserving ecosystem C storage under grazing disturbances. It is essential that future management strategies incorporate these aspects to effectively protect and enhance C sequestration in alpine meadows.

## 1 Introduction

Grasslands represent the largest terrestrial ecosystem globally, covering approximately 40% of the Earth’s land surface (Bai and Cotrufo, 2022). However, in recent years, over 70% of ecosystems have degraded due to overgrazing and climate change, resulting in disruptions to the carbon (C) cycles and declines in ecosystem services (Xu et al., 2013). The Tibetan Plateau, as the largest alpine ecological barrier globally, not only safeguards regional ecological security but also constitutes a major carbon reservoir. However, degradation pressures are also substantial, posing challenges to the stability of carbon stocks and the system’s recovery capacity (Dai et al., 2021). To address this issue, the government has implemented banned grazing and rest grazing policies, aiming to balance livestock development with ecological conservation (Huang et al., 2021).

Current policy assessments predominantly focus on macroscopic changes in C storage and there is a lack of mechanistic understanding of how grazing patterns regulate the C: nitrogen (N): phosphorus (P) stoichiometry of vegetation and thereby influence ecosystem C pools. Numerous studies have revealed systematic effects of grazing patterns on ecosystem C storage: moderate grazing may enhance ecosystem C storage in alpine meadow ecosystems (Wang et al., 2024), and long-term banned grazing (BG) have also been reported to substantially improve ecosystem C storage (Dai et al., 2021). However, these conclusions remain uncertain across different regions, scales, and study designs. Additionally, mechanistic studies on the relationship between grazing and C storage require systematic quantification.

Within plant functional groups, several trend-like findings emerge for the C: N: P stoichiometry. As grazing intensity increases, the C: N ratio in grasses declines, while the N: P ratio rises. Grasses generally exhibit higher C content and C: N ratios compared to legumes, whereas legumes showed higher N: P ratios than grasses and other functional groups (Wang et al., 2022a). Across Chinese grasslands, legumes exhibit significantly higher leaf N: P ratios than grasses and other plant functional groups (He et al., 2008; Zhang et al., 2010). This finding suggested that the differences in stoichiometric traits among functional groups may operate through the allocation of C, N, and P within plant tissues to influence ecosystem C storage. However, current studies have focused on isolated analyses of either ecosystem C storage or plant functional group stoichiometry (Wang et al., 2024, 2022a), lacking systematic examination of the coupling relationship between grazing patterns, functional group stoichiometry ratios, and ecosystem C storage.

Therefore, this study aims to systematically investigate the distribution patterns of vegetation C: N: P stoichiometry and its coupling mechanisms with ecosystem C storage under different grazing patterns [BG, growing season rest-grazing (RG), traditional rest-grazing (TG), and the continuous grazing (CG)] on the Tibetan Plateau. By integrating multi-compartment data across the vegetation (plant functional groups)-litter-soil continuum, we focus on elucidating two key scientific questions: (1) How do grazing patterns influence C, N, and P concentrations and C: N: P stoichiometry in vegetation components? (2) How do plant functional group biomass and C: N: P stoichiometry regulate ecosystem C storage? The findings will provide critical theoretical support for adaptive management of alpine grasslands and offer practical implications for sustainable grassland resource utilization under China’s “carbon peaking” and “carbon neutrality” goals.

## 2 Materials and methods

### 2.1 Study area

Situated at an altitude of roughly 2960 meters, the study area lies along the northeastern margin of the Tibetan Plateau within the Jinqiang River region, Tianzhu County. Its coordinates are approximately 36°31′ - 37°55′N and 102°07′ - 103°46′E (**Supplementary Figure S1**). This region experiences a chilly and moist climate shaped by a continental plateau monsoon. The average annual temperature is approximately 0.16°C, with total yearly precipitation nearing 416.9 mm. Plants have a growing season that lasts about 120 to 140 days. The soil found here is classified as alpine chernozem, and the type of grassland present is an alpine meadow (He et al., 2025). The main dominant species of the grasses are *Poa pratensis*, *Elymus nutans*. The main dominant species of the sedges are *Kobresia capillifolia* and *K. pygmaea*. The main dominant species of the legumes are *Melissitus ruthenica*, *Oxytropis ochrocephala*. The main dominant species of forbs include species such as *Bistorta vivipara* and *Potentilla chinensis* et al.

### 2.2 Experiment design

The location chosen for this analysis is an alpine meadow that is moderately deteriorated (Li, 1997), which participated in the “Returning Grazing Land to Grassland” initiative launched in 2003. Throughout the duration of the project, the natural features of the site, such as terrain, slope orientation, and gradient, stayed unchanged. Indigenous Tibetan sheep and yaks were the grazing animals found in this region. In 2003, a study was initiated to examine the effects of different grazing patterns on degraded alpine meadow plots. The four patterns implemented were banned grazing (BG), growing season rest-grazing (RG), traditional rest-grazing (TG), and continuous grazing (CG). The research employed a randomized block design that comprised three distinct block groups. Within each block group, four separate treatment plots were established, leading to a total of twelve plots, each measuring 100 m by 100 m. The grazing patterns were clearly defined to assess their specific impacts on the meadows. The BG involved the prohibition of all grazing for the entire year, which effectively meant a stocking rate of 0 sheep units per hectare annually. In contrast, the RG allowed grazing from late April until the end of September, maintaining a moderate stocking rate of 3.07 sheep units per hectare each year. The TG permitted grazing only from late June to the end of September, with a slightly higher stocking rate set at 4.60 sheep units per hectare annually. Lastly, the CG allowed for unrestricted grazing throughout the entire year, with the highest stocking rate of 6.13 sheep units per hectare per year. This structured approach was instrumental in analyzing how varied grazing regulations influenced the meadow’s ecological health over time (He et al., 2024).

### 2.3 Community survey and soil sample collection

In August 2022, we conducted a survey of the plant community and collected soil samples. Within every sample plot, three quadrats (1 m × 1 m) were placed at random locations, ensuring that there was at least a 50 m separation between each quadrat. As a result, we gathered a total of 36 quadrats (3 blocks × 4 treatments × 3 quadrats) (He et al., 2025).

Based on preliminary data showing that root depth typically did not exceed 40 cm, a 25 cm × 25 cm × 40 cm soil cube was collected in each 1 m × 1 m quadrats, capturing the aboveground and belowground biomass. To ensure root integrity and cleanliness, each soil cube sample was carefully rinsed multiple times until attached soil and dead roots were completely removed. After cleaning, the washed plant individuals was classified into functional groups: grasses, sedges, legumes, and forbs. Each plant individual was separated into aboveground and root components. All aboveground, and root samples were killed at 105°C for 30 minutes, and subsequently dried at 75°C for 72 hours to determine their biomass.

Litter collection was conducted within each 1 m × 1 m quadrats by mowing to collect aboveground senescent stems and leaves. It was air-dried in a cool, shaded area, then screened to remove gravel and impurities and stored in nylon bags for subsequent analysis.

Concurrently, soil sampling and vegetation survey quadrats were conducted at the same locations. A soil auger (Φ = 38 mm) was employed to obtain stratified soil samples from topsoil (0–20 cm) and subsoil (20–40 cm) intervals. Soil samples were obtained through the “five-point method”. The soil bulk density (BD) was assessed using soil ring. Following air-drying, soil samples underwent purification by removing root materials and extraneous debris, then underwent sieving through a 2 mm aperture mesh as part of the pre-analytical preparation for soil C concentration determination (Zhang et al., 2022c).

### 2.4 Nutrient determination and calculation

#### 2.4.1 Determination of vegetation nutrient content, calculation of vegetation nutrient storage, and C: N: P stoichiometry

Dried vegetation samples (aboveground, roots and litter) were ground and C and N concentrations in the plants were analyzed by a fully automated carbon and nitrogen analyzer (Primacs SNC 100-IC-E; Sklar, The Netherlands) (He et al., 2024). P concentration was measured through H_2_SO_4_-H_2_O_2_ digestion, followed by analysis using the molybdenum-antimony colorimetric technique (Parkinson and Allen, 1975). The concentration of potassium (K) was assessed through H_2_SO_4_-H_2_O_2_ digestion and subsequent analysis by flame photometry (Parkinson and Allen, 1975).

C, N, P, and K concentrations (g·kg^-1^) of aboveground vegetation, roots and litter were multiplied by the biomass of the corresponding fractions (kg·m^-2^) to derive the C, N, P, and K storage of each of their fractions (g·m^-2^) (Cao and Chen, 2017).

In this study, the C: N: P stoichiometry is calculated based on element concentrations.

#### 2.4.2 Soil nutrient determination and carbon storage calculation

The concentration of soil C was measured using the heating technique involving potassium dichromate and sulfuric acid. To estimate soil C concentration in various layers of a 40 cm soil profile, the “equivalent soil mass” approach is typically employed, assessing each 20 cm section (Liu et al., 2022; He et al., 2024).


$${\text{M}}_{soil,\;i}(\text{Mg}\cdot {\text{hm}}^{-2})={\text{BD}}_{i}\times {\text{T}}_{i}\times 10000$$

$${\text{SC}}_{\text{storage}}(\text{g}\cdot {\text{m}}^{-2})=\sum_{i=1}^{\text{n}}[{\text{M}}_{soil,\;i}\times {\text{C}}_{i}+({\text{M}}_{o,i}-{\text{M}}_{soil,\;i})\times {\text{C}}_{i+1}]\times 0.1$$

Where, SC _storage_ is the soil C storage. M *_soil_*, *_i_* is the soil mass of the *i* th layer (Mg·hm^-2^). BD*_i_* and T*_i_* are the bulk density (g·cm^-^³) and thickness (m) of the *i* th layer. C*_i_* and C*_i+_*_1_ are the soil C concentrations in the *i* th and *i+*1 th layers (g·kg^-^¹). M*_o_*, *_i_* is the maximum soil mass from the first to the *n* th layer (He et al., 2024).

#### 2.4.3 Calculation of ecosystem carbon storage

This study employed a direct summation method, calculating the total ecosystem carbon storage by summing aboveground biomass carbon, root carbon, litter carbon, and soil carbon storage (Lu et al., 2015).

### 2.5 Data analysis

To test whether there are significant differences in the concentrations and storage of C, N, P, and K in aboveground vegetation, roots, and litter, as well as the ratios of C: N, C: P, N: P and ecosystem C storage across different grazing patterns and functional groups. Normality and homogeneity of variances were assessed for all variables (Zhang et al., 2023), and a one-way analysis of variance (ANOVA) was then performed (Fox and Weisberg, 2019). The model includes both treatment (treat) and block factors to account for variability among blocks. Convert variables to natural logarithms if necessary. When differences were significant, a *post hoc* test utilizing the least significant difference (LSD) method was employed to detect differences among grazing patterns and functional groups. Statistical analyses were conducted with SPSS software (version 26.0), and significance was determined at the α = 0.05 level. Pearson correlation analysis was conducted using the ‘corrplot’ R package to assess the relationships between ecosystem C storage and plant functional group aboveground biomass (AGB), root biomass (BGB), litter, and vegetation C: N: P stoichiometry. Subsequently, random forest analysis was performed with the ‘randomForest’ R package to identify the primary influencing factors of ecosystem C storage. Finally, Partial Least Squares Path Modeling (PLS-PM) was employed to explore the potential associations between grazing patterns, aboveground stoichiometry, functional group biomass, and ecosystem C storage, utilizing the ‘plspm’ R package.

## 3 Results

### 3.1 Variations in C, N, P, and K concentrations of aboveground vegetation, roots, and litter across different grazing patterns

Different grazing patterns significantly affected the concentrations of C, N, P, and K in aboveground biomass, roots, and litter (**Tables 1**, **2**). Under the RG pattern, the highest concentrations of C, P, and K were observed in the aboveground parts of sedges and forbs, while grasses maintained relatively high levels. In roots, C and P concentrations were highest across sedges and legumes under RG pattern. Additionally, the concentrations of C, N, P, and K in litter under the RG pattern were higher than those in other patterns, with mean values of 398.75 g·kg^−1^ for C, 23.10 g kg^−1^ for N, 2.33 g·kg^−1^ for P, and 18.68 g·kg^−1^ for K. Among functional groups, legumes exhibited significantly higher N concentrations in the aboveground parts and roots than others, and root P concentrations also remained at elevated levels. Each plant functional group exhibited specific responses to different grazing patterns, with legumes showing a pronounced advantage in the N cycle.

**Table 1:** Vegetation C and N concentrations under different grazing patterns.

Ecosystem components		C concentration (g kg^−1^)				N concentration (g kg ^−1^)			
		BG	RG	TG	CG	BG	RG	TG	CG
Aboveground	Grasses	403.62 ± 4.89^Aa^	395.62 ± 5.13^Ab^	391.79 ± 5.59^Aa^	388.14 ± 5.80^Aa^	20.95 ± 0.35^Dc^	22.34 ± 0.35^Cc^	23.66 ± 0.55^Bc^	25.30 ± 0.30^Ac^
	Sedges	390.28 ± 4.34^ABa^	399.70 ± 1.90^Aab^	381.96 ± 5.36^Ba^	364.90 ± 5.77^Ca^	21.72 ± 0.29^Cc^	21.60 ± 0.16^Cc^	23.11 ± 0.43^Bc^	24.40 ± 0.16^Ac^
	Legume	390.07 ± 5.77^ABa^	410.82 ± 4.93^Aa^	289.20 ± 8.90^Cb^	369.26 ± 13.61^Ba^	28.32 ± 0.35^Ba^	28.02 ± 0.31^Ba^	29.50 ± 0.99^ABa^	30.66 ± 0.49^Aa^
	Forbs	401.11 ± 6.28^ABa^	409.20 ± 3.79^Aa^	393.94 ± 4.50^BCa^	380.08 ± 5.28^Ca^	23.15 ± 0.60^Cb^	24.33 ± 0.26^Cb^	25.70 ± 0.45^Bb^	27.28 ± 0.56^Ab^
Root	Grasses	380.10 ± 6.54^Bb^	409.21 ± 2.19^Aab^	414.09 ± 5.36^Aa^	413.32 ± 5.68^Aa^	18.12 ± 0.54^Ab^	17.48 ± 0.53^Ab^	17.79 ± 0.49^Ab^	17.67 ± 0.53^Ac^
	Sedges	393.07 ± 3.69^Ab^	402.18 ± 3.43^Ab^	398.00 ± 4.24^Ab^	394.11 ± 7.40^Ab^	17.74 ± 0.40^Bb^	18.97 ± 0.45^Aab^	18.73 ± 0.62^ABb^	18.46 ± 0.32^ABbc^
	Legume	413.68 ± 7.14^Aa^	413.88 ± 2.39^Aa^	409.90 ± 3.67^Aab^	404.97 ± 4.73^Aab^	23.77 ± 0.76^Aa^	20.56 ± 0.98^Ba^	21.69 ± 0.79^ABa^	22.23 ± 0.50^ABa^
	Forbs	413.46 ± 3.65^Aa^	412.41 ± 3.14^Aa^	407.32 ± 4.34^Aab^	402.56 ± 6.94^Aab^	18.11 ± 0.55^Bb^	19.11 ± 0.70^ABab^	19.29 ± 0.54^ABb^	19.53 ± 0.37^Ab^
Litter		387.00 ± 4.02^A^	398.75 ± 3.87^A^	356.64 ± 6.06^B^	368.28 ± 6.72^B^	21.07 ± 0.76^B^	23.10 ± 0.44^A^	21.77 ± 0.69^AB^	21.18 ± 0.70^B^

Capital letters signify significant variations among various treatments within a single functional group, whereas lowercase letters denote significant differences among different functional groups under the same treatment (*P* < 0.05, *n* = 9).

**Table 2:** Vegetation P and K concentrations under different grazing patterns.

Ecosystem components		P concentration (g kg ^−1^)				K concentration (g kg ^−1^)			
		BG	RG	TG	CG	BG	RG	TG	CG
Aboveground	Grasses	1.77 ± 0.06^Cc^	2.55 ± 0.12^Aab^	2.19 ± 0.08^Ba^	1.54 ± 0.02^Db^	16.29 ± 0.32^Ac^	16.76 ± 0.28^Ac^	15.49 ± 0.23^Bc^	14.80 ± 0.16^Bc^
	Sedges	1.90 ± 0.04^Bbc^	2.36 ± 0.12^Ab^	1.81 ± 0.04^Bb^	1.59 ± 0.04^Cb^	15.80 ± 0.40^Ac^	15.81 ± 0.21^Ad^	15.18 ± 0.15^Ac^	14.27 ± 0.29^Bc^
	Legume	2.06 ± 0.03^BCa^	2.61 ± 0.15^Aab^	2.23 ± 0.08^Ba^	1.86 ± 0.06^Ca^	19.72 ± 0.59^Ab^	19.55 ± 0.38^Ab^	17.42 ± 0.20^Bb^	16.48 ± 0.36^Bb^
	Forbs	1.94 ± 0.05^Cab^	2.78 ± 0.11^Aa^	2.14 ± 0.07^Ba^	1.87 ± 0.07^Ca^	21.56 ± 0.38^Ba^	22.89 ± 0.41^Aa^	21.44 ± 0.21^Ba^	20.85 ± 0.30^Ba^
Root	Grasses	1.99 ± 0.07^Bb^	2.31 ± 0.09^Ab^	2.00 ± 0.04^Bb^	1.79 ± 0.07^Cb^	4.30 ± 0.16^Ab^	3.90 ± 0.14^Ac^	3.38 ± 0.14^Bc^	2.84 ± 0.20^Cb^
	Sedges	2.12 ± 0.07^Bb^	2.37 ± 0.11^Ab^	2.07 ± 0.06^Bb^	1.75 ± 0.07^Cb^	4.39 ± 0.26^Ab^	3.60 ± 0.13^Bc^	3.00 ± 0.11^Cc^	2.84 ± 0.16^Cb^
	Legume	2.62 ± 0.13^ABa^	2.94 ± 0.23^Aa^	2.27 ± 0.06^BCa^	2.07 ± 0.09^Ca^	7.28 ± 0.42^Aa^	6.74 ± 0.38^ABa^	7.19 ± 0.20^Aa^	5.99 ± 0.28^Ba^
	Forbs	2.46 ± 0.09^Aa^	2.68 ± 0.09^Aab^	2.23 ± 0.05^Ba^	1.97 ± 0.07^Cab^	4.69 ± 0.13^BCb^	4.92 ± 0.19^Bb^	4.09 ± 0.28^Cb^	6.55 ± 0.22^Aa^
Litter		1.93 ± 0.08^BC^	2.33 ± 0.12^A^	1.95 ± 0.05^B^	1.64 ± 0.14^C^	18.49 ± 0.49^A^	18.68 ± 1.20^A^	17.22 ± 0.60^AB^	16.22 ± 0.35^B^

Capital letters signify significant variations among various treatments within a single functional group, whereas lowercase letters denote significant differences among different functional groups under the same treatment (*P* < 0.05, *n* = 9).

### 3.2 Variations in C: N: P stoichiometry of aboveground vegetation, roots, and litter across different grazing patterns

**Figure 1:**
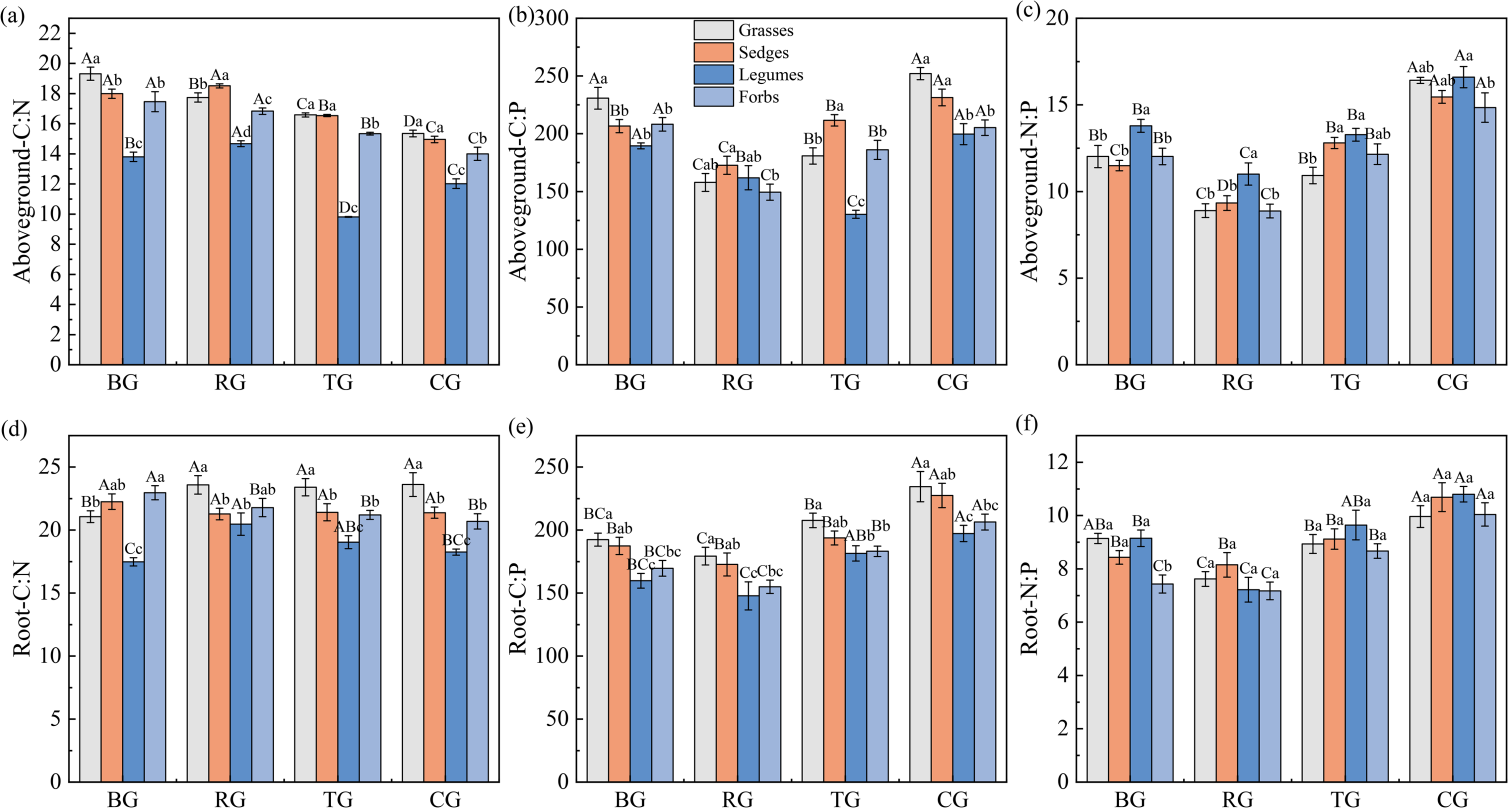
Ratios of C: N **(a, d)**, C: P **(b, e)**, and N: P **(c, f)** in aboveground and root under different grazing patterns. Capital letters signify significant variations among various treatments within a single functional group, whereas lowercase letters denote significant differences among different functional groups under the same treatment (*P* < 0.05, *n* = 9).

Grazing patterns significantly altered the C: N: P stoichiometry of plant functional groups in both aboveground vegetation and roots (**Figure 1**). Aboveground, the C: N ratios were highest in grasses and forbs under the BG pattern, with a decreasing trend following BG > RG > TG > CG (**Figure 1a**). Except for forbs, the C: P ratios of other functional groups under CG were significantly higher than those in other patterns (*P* < 0.05, **Figure 1b**). All four functional groups exhibited significantly higher N: P ratios under CG compared to BG, TG, and RG (*P* < 0.05, **Figure 1c**). Root responses varied: grasses and legumes exhibited the lowest C: N ratios under BG, whereas sedges and forbs showed the opposite pattern (**Figure 1d**). The root C: P and N: P ratios peaked under CG across all functional groups (**Figures 1e-f**). Plant functional group comparisons revealed that legumes had the lowest aboveground C: N and the highest N: P ratios, whereas grasses exhibited higher root C: P ratios than other functional groups.

**Figure 2:**
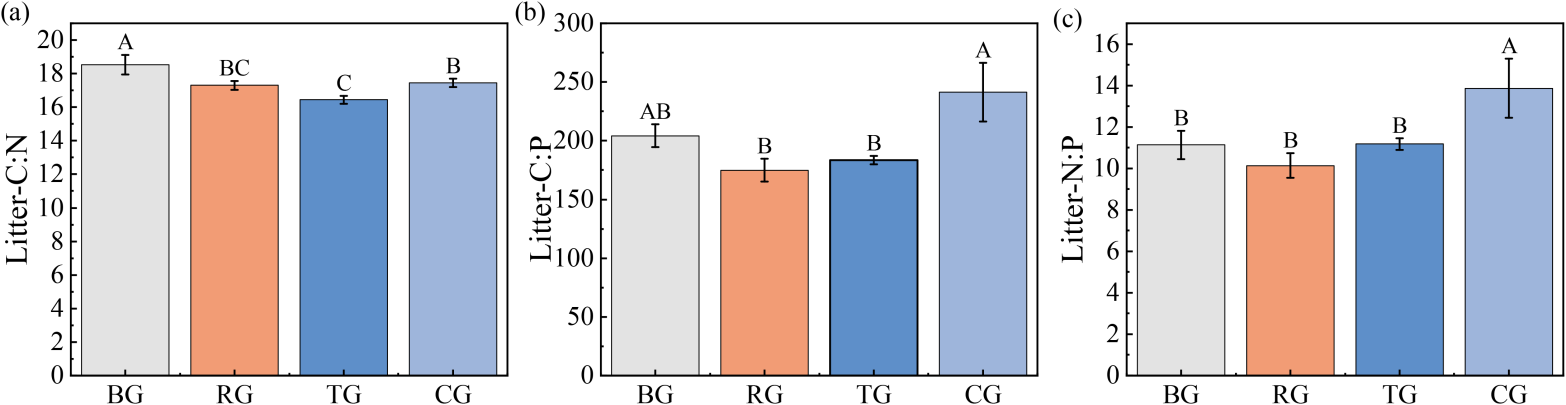
Ratios of C: N **(a)**, C: P **(b)**, and N: P **(c)** in litter under different grazing patterns. Distinct capital letters designate statistically significant differences among various treatments (*P* < 0.05, *n* = 9).

Analyses in **Figure 2** show litter C: N: P stoichiometry changes across grazing patterns: the litter C: N ratio was significantly higher under BG than under RG, TG, and CG (**Figure 2a**). **Figures 2b, c** indicate that the CG pattern produced the peak litter C: P and N: P ratios, whereas RG showed the lowest values.

### 3.3 Variations in C, N, P, and K storage of aboveground vegetation, roots, and litter across different grazing patterns

**Figure 3:**
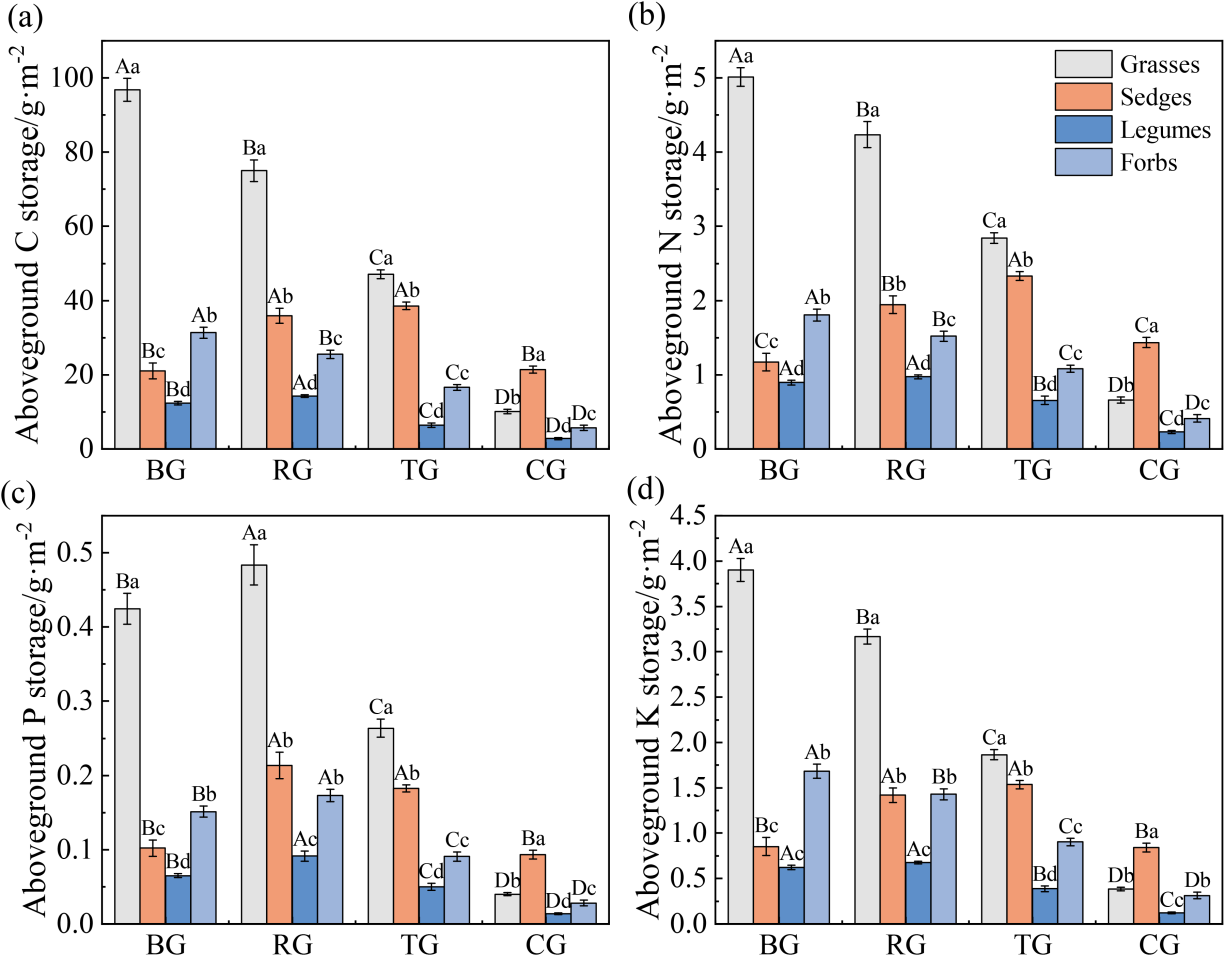
Aboveground C **(a)**, N **(b)**, P **(c)**, and K **(d)** storage of functional groups under different grazing patterns. Capital letters signify significant variations among various treatments within a single functional group, whereas lowercase letters denote significant differences among different functional groups under the same treatment (*P* < 0.05, *n* = 9).

Grazing patterns significantly altered the aboveground storage of C, N, P, and K across plant functional groups (**Figure 3**, *P* < 0.05). Aboveground storage of C, N, and K in grasses and forbs followed a gradient: BG > RG > TG > CG. In sedges, aboveground storage of C, N, and K was significantly higher under the TG and RG patterns compared to the CG and BG patterns. Legumes showed peak aboveground storage of C, N, and K under the RG pattern (**Figures 3a, b, d**). RG exhibited the highest aboveground P storage across all functional groups, significantly exceeding BG and CG (*P* < 0.05, **Figure 3c**). Grasses dominated aboveground C, N, P and K storage under BG, RG and TG, whereas sedges attained the highest values under CG (*P* < 0.05).

**Figure 4:**
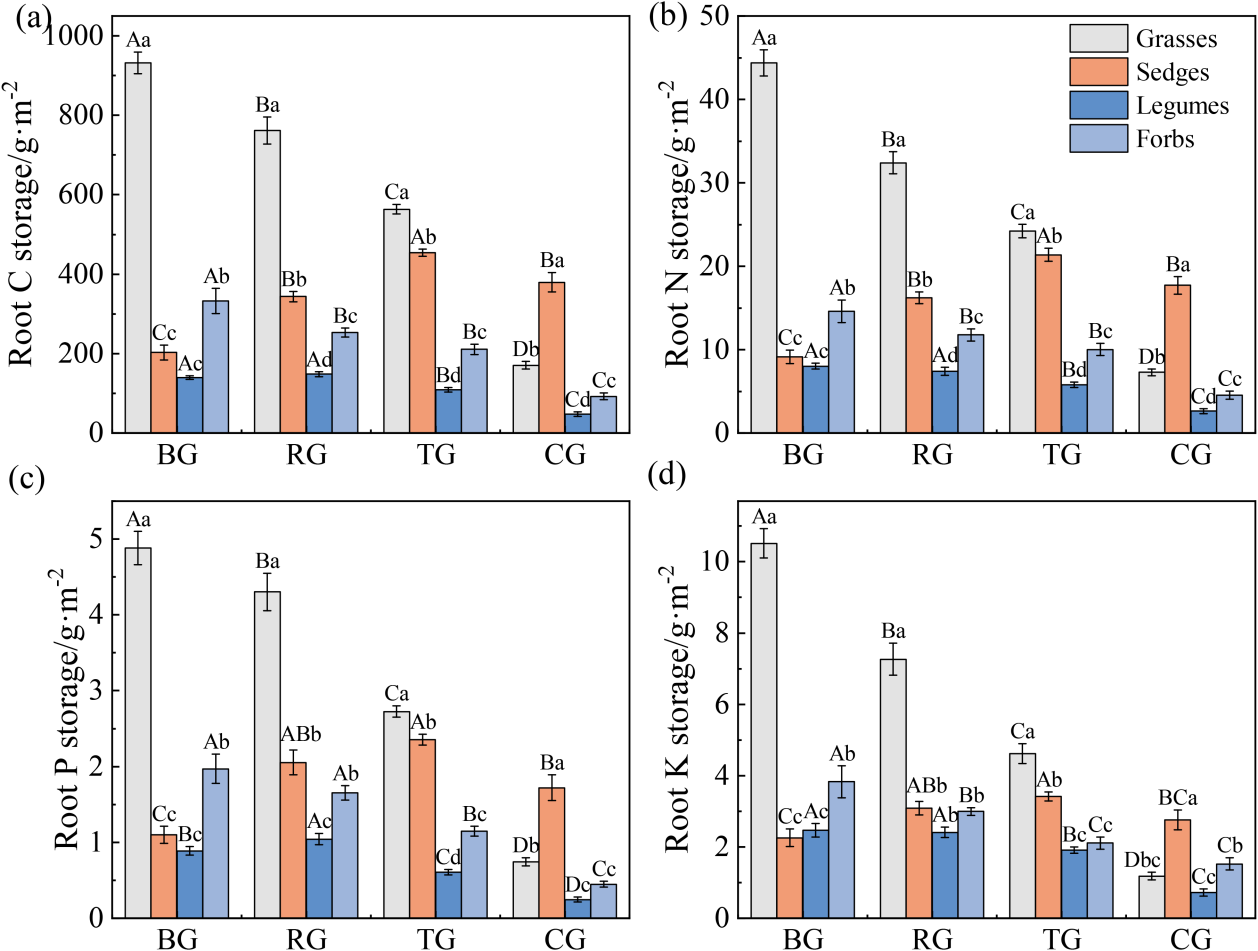
Root C **(a)**, N **(b)**, P **(c)**, and K **(d)** storage of functional groups under different grazing patterns. Capital letters signify significant variations among various treatments within a single functional group, whereas lowercase letters denote significant differences among different functional groups under the same treatment (*P* < 0.05, *n* = 9).

Grazing patterns significantly affected root C, N, P and K storage (**Figures 4a-d**, *P* < 0.05). Root C, N, P, and K storage of grasses and forbs followed a gradient of BG > RG > TG > CG. Root nutrient storage in sedges peaked under TG, whereas those in legumes were lowest under CG (*P* < 0.05). Grasses consistently exhibited the highest root nutrient storage under BG, RG, and TG (*P* < 0.05), but sedges surpassed all other functional groups under CG (*P* < 0.05).

**Figure 5:**
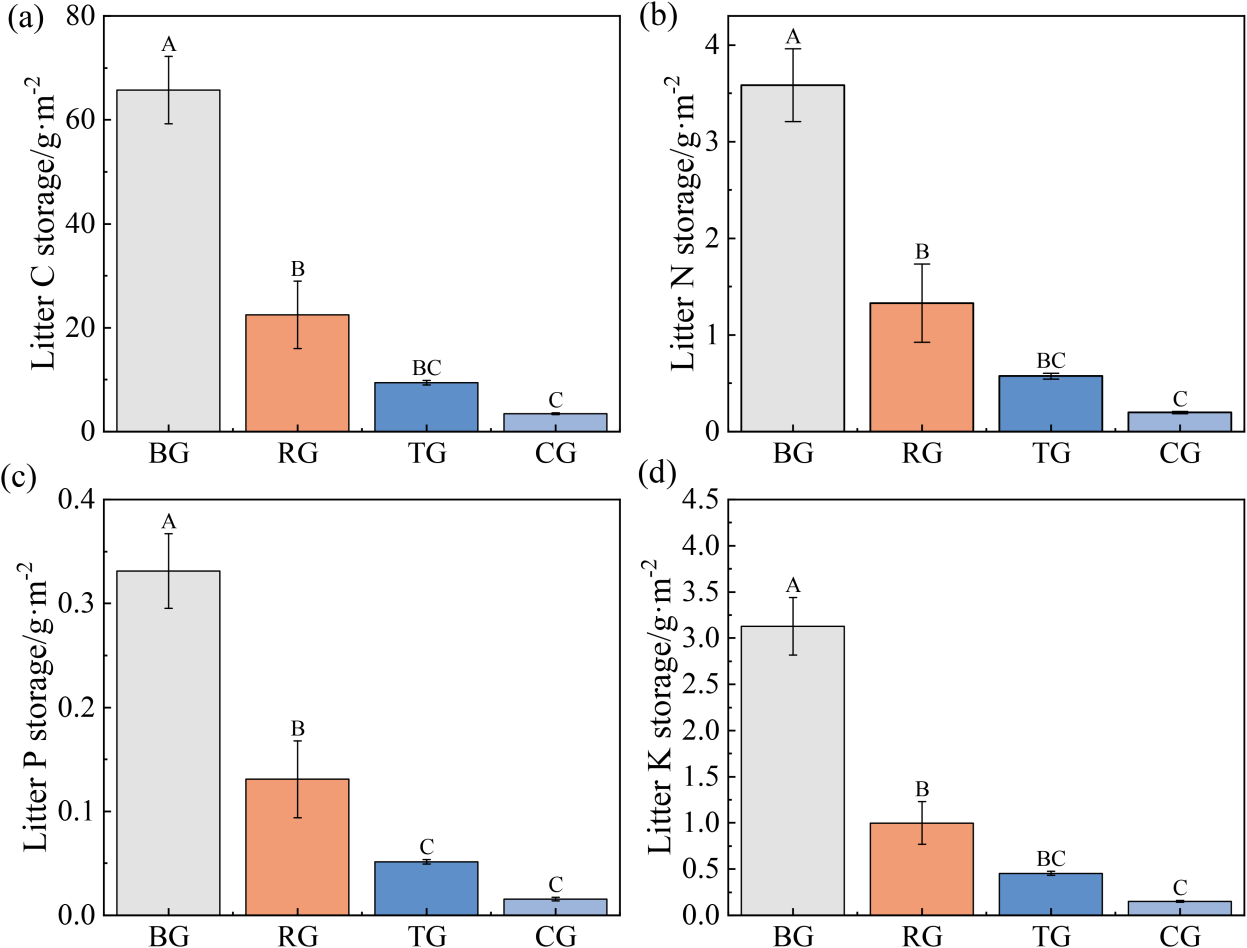
Litter C **(a)**, N **(b)**, P **(c)**, and K **(d)** storage under different grazing patterns. Distinct capital letters designate statistically significant differences among various treatments (*P* < 0.05, *n* = 9).

Litter C, N, P and K storage under BG exceeded those of all other patterns, whereas TG and CG did not significant differences (**Figures 5a-d**).

### 3.4 Ecosystem carbon storage and allocation patterns in alpine meadows under different grazing patterns

**Figure 6:**
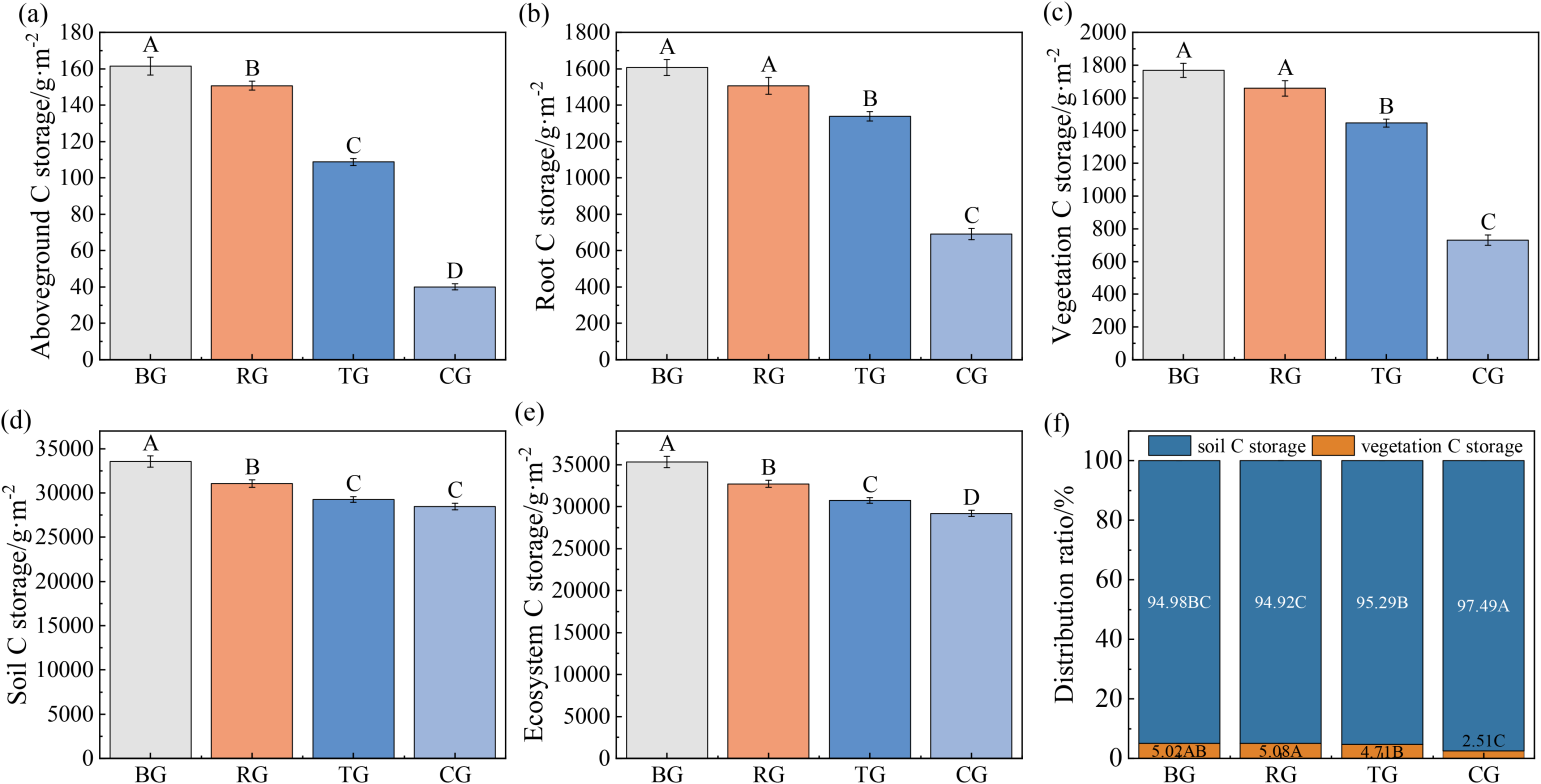
Ecosystem C storage **(a-e)** and distribution patterns **(f)** of alpine meadow under different grazing patterns. Distinct capital letters designate statistically significant differences among various treatments (*P* < 0.05, *n* = 9).

Different grazing patterns significantly affected the ecosystem C storage of alpine meadows, following the trend: BG > RG > TG > CG (**Figures 6a-e**, *P* < 0.05). Soil C storage (28447.22–33545.78 g·m^-^²) comprised 94.92–97.49% of total ecosystem C storage (29177.72–35314.13 g·m^-^²), whereas vegetation contributed only 2.51–5.08% (**Figure 6f**).

### 3.5 Factors influencing ecosystem C storage

**Figure 7:**
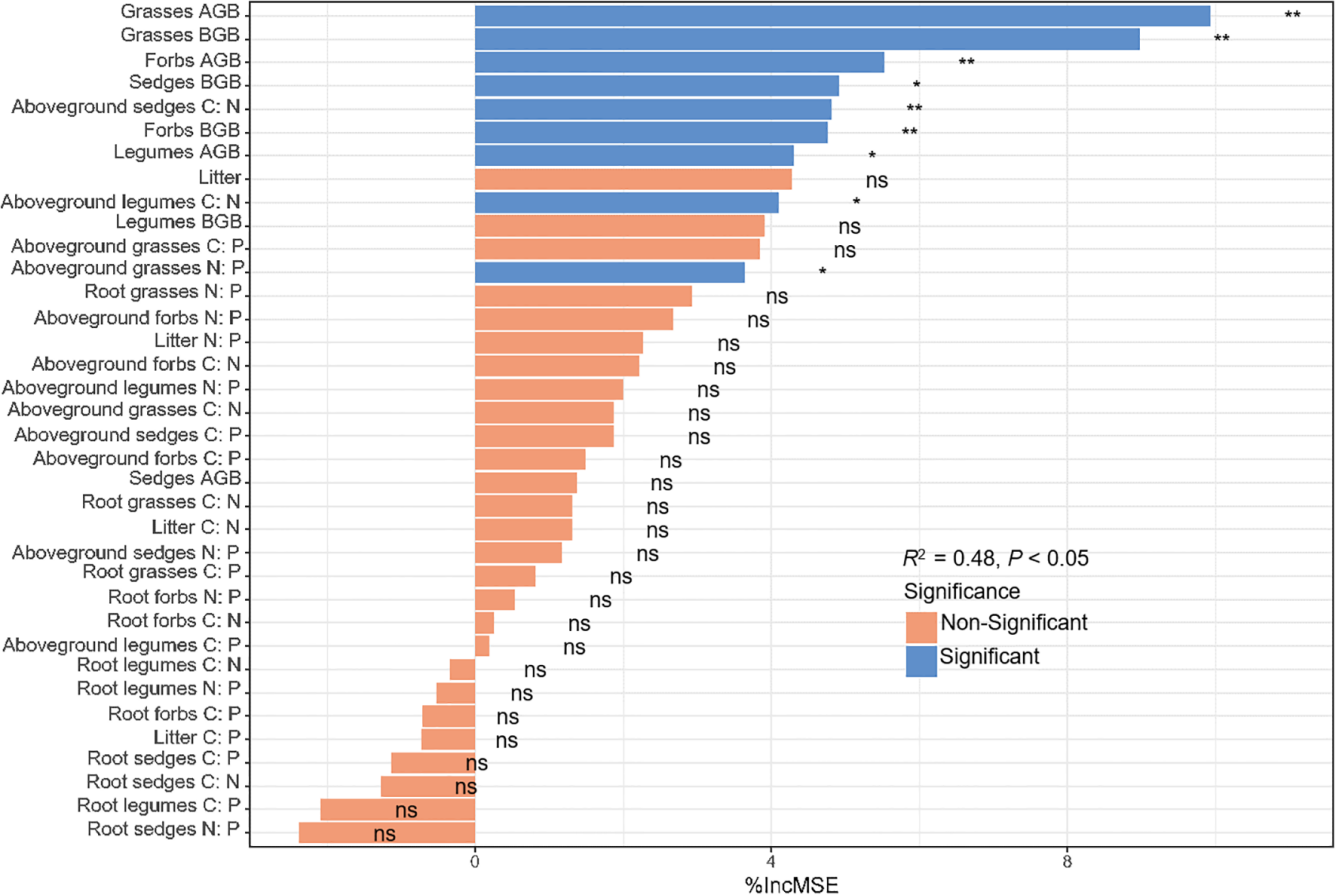
Relative importance of vegetation, root biomass and C: N: P stoichiometry for ecosystem C storage. *P < 0.05; **P < 0.01.

Ecosystem C storage correlated strongly with plant biomass and C: N: P stoichiometry (**Supplementary Figure S2**). Random forest modelling showed that AGB, BGB, litter biomass and C: N: P stoichiometry collectively explained 48% of its variance. The most influential predictors were grasses AGB and BGB, forbs AGB, sedges C: N ratios and forbs BGB (*P* < 0.01), whereas significant effects were also observed for sedges BGB, legumes AGB, legumes aboveground C: N and grasses N: P ratios (*P* < 0.05, **Figure 7**).

**Figure 8:**
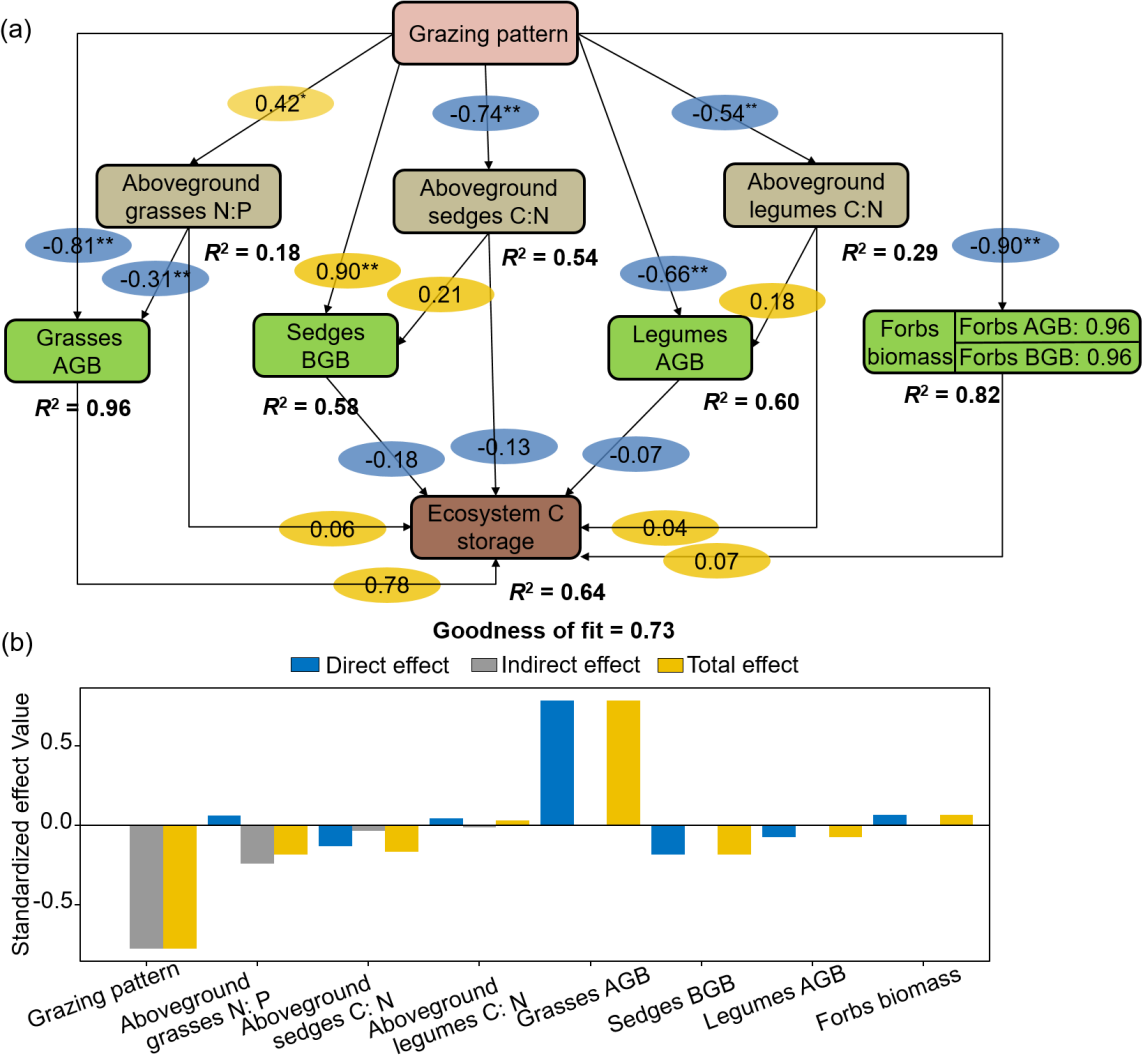
Structural equation modelling of grazing patterns, aboveground C: N: P stoichiometry and aboveground, root biomass to ecosystem C storage **(a)** and standardized effect values **(b)**.

PLS-PM showed that grazing patterns indirectly influenced ecosystem C storage by plant aboveground stoichiometry and functional group biomass (**Figure 8a**). Aboveground grasses N: P and legumes C: N ratios positively affected ecosystem C storage directly or indirectly through biomass. Ecosystem C storage was directly and positively affected by grasses and forbs AGB, while was negatively impacted by sedges BGB and legumes AGB. The standardized total effect of grasses AGB on ecosystem C storage was 0.78 (**Figure 8b**).

## 4 Discussion

### 4.1 Impact of grazing patterns on vegetation nutrient concentration and C: N: P stoichiometry

C, N, P, and K are essential nutrients for plants and significantly influence their growth and ecosystem functions (Fang et al., 2024). Vegetation C and K concentrations peaked under RG indicated that moderate disturbance accelerates C fixation and K acquisition in alpine meadows. Grazing (BG-CG) induced changes in plant community structure, such as increased legumes abundance, led to a gradual increase in aboveground N concentrations, while both aboveground and root P concentrations decreased. For example, under the CG pattern, increased nitrogen from fecal input enhanced plant N absorption (Liu et al., 2004), whereas soil P content declined, reducing P accumulation in plants (Liu et al., 2021a). These findings are consistent with Wang et al. (2022a), who reported similar trends in the Inner Mongolia alpine steppe. There are significant differences in nutrient demand and uptake among plant functional groups, legumes exhibited the highest N and P concentrations, and their roots contained higher N, P, and K levels, indicating strong N fixation capacity and less N limitation (Kebede, 2021). Meanwhile, in aboveground grasses, C concentrations were higher under BG than CG. These variations reflect selective nutrient absorption by plants and the nutrient limitations faced by different functional groups (Reich and Oleksyn, 2004; Liu et al., 2021b; Zhang et al., 2022a). Future work should use organ and compound level analyses to deepen understanding of how different plant functional groups respond to grazing, thereby enhancing insights into grazing impacts on alpine meadow ecosystems.

Vegetation stoichiometry are influenced by multiple factors, including species composition, soil moisture and nutrients, temperature, and light conditions, as well as the interactions among these factors (Zhang et al., 2022b). The N: P ratio remains a widely used indicator of nutrient limitation, with thresholds of <14 for N limitation, >16 for P limitation, and 14–16 for co-limitation (Güsewell, 2004). Our results indicated that under the CG pattern, the N: P ratios of various functional groups suggested a joint limitation of N and P in plant nutrition. Under the other grazing patterns, the N: P ratios of aboveground, roots, and litter mainly reflected N limitation. These findings align with Zhang et al. (2014), who concluded that plant communities in Tibetan Plateau alpine meadows are primarily N-limited by limiting factor analysis. Across plant functional groups, C: N: P stoichiometry varied markedly, with forbs generally displaying lower C: N and C: P ratios in both aboveground and roots than grasses and sedges under CG patterns, suggesting faster growth rates for forbs relative to grasses and sedges. These patterns may reflect differential nutrient use strategies among functional groups and could influence competitive dynamics under grazing treatments. Specifically, fast-growing forbs may dominate the community, while slower-growing grasses and sedges may be gradually crowded out due to their weaker competitiveness, ultimately reducing forage production in alpine meadows and potentially limiting the development of local livestock (Wang et al., 2022b). Compared with other plant functional groups, legumes did not show a consistent pattern in growth rates, but consistently exhibited the highest N: P ratios in aboveground across four grazing patterns, suggesting a greater susceptibility of legume growth to P limitation. This observation aligns with He et al. (2008), who reported significantly higher N: P ratios in legumes than in grasses, sedges, and forbs. Differences in C: N: P stoichiometry among functional groups reflect the variability in their nutrient limitation states, which may contribute to the maintenance of biodiversity.

### 4.2 Impact of grazing patterns on vegetation’s nutrient storage

Vegetation nutrient storage studies demonstrate significant variability in results, even among similar regions, primarily due to heterogeneity in vegetation distribution and differences in statistical analysis methods. This study quantified the storage of C, N, P, and K in aboveground biomass, roots, and litter across various grazing patterns. The aboveground C storage exhibited significant variability, with N, P, and K showing similarly broad ranges. Root storage for C, N, P, and K also demonstrated considerable differences, while litter storage of C, N, P, and K reflected notable trends. These results highlight the impact of grazing patterns on nutrient element storage. Furthermore, the relationship between vegetation nutrient element storage, and concentration, biomass complicates how grazing patterns impact nutrient storage in vegetation. This study revealed that grasses and forbs exhibited the highest C, N, and K storage under the BG pattern, while sedges demonstrated peak C, N, and K storage under the TG pattern. In contrast, legumes showed the greatest levels of C, N, P, and K storage under the RG pattern. These results suggest that effective vegetation management must consider the distinct reactions of different plant groups to grazing intensity. Specifically, from BG to CG, nutrient storage in grasses and forbs may gradually decrease, while sedges and legumes might first increase before decreasing. The findings indicated that grazing patterns significantly influence community composition and nutrient storage distribution among functional groups. The BG, RG, and TG patterns enhanced nutrient storage in grasses, while the CG pattern notably boosted nutrient storage in legumes. This aligns with previous studies indicating that grazing patterns can alter community composition and structure. For instance, Song et al. (2020) found that after 20 years of grazing prohibition in Qinghai Haibei, C storage in grasses increased while sedges decreased, supporting our results and indicating that grasses dominance significantly influences nutrient storage dynamics.

Plant roots are crucial for C storage, as their biomass and nutrient concentration affect overall nutrient storage in vegetation (Tariq et al., 2024). Variability in climatic and environmental conditions, grazing patterns, and plant traits leads to differences in root number and distribution, resulting in significant variations in root nutrient storage across grazing patterns (Sun et al., 2021). This study found that roots of grasses and forbs under the BG grazing pattern had significantly higher C, N, P, and K storage compared to other grazing patterns (*P* < 0.05), consistent with previous literature (Li et al., 2018; Wang et al., 2022a). This phenomenon may arise from the significant impact of grazing patterns on the biomass and productivity of plant roots (Dai et al., 2019). Under the BG pattern, where grazing disturbance is absent, grasses and forbs grow rapidly, resulting in higher root productivity. Meanwhile, differences in root nutrient concentrations among treatments were small compared to root biomass. Consequently, root nutrient storage of grasses and forbs was relatively high in the BG pattern. Additionally, this study found that sedge roots had the highest nutrient storage under the TG pattern, likely due to their responsiveness to soil environmental conditions. Plant roots were primarily distributed in the topsoil, where water and nutrient availability is better, while grazing patterns influenced the spatial variability of soil nutrient utilization (Li et al., 2017). There is spatial variability in root biomass and nutrient storage among different functional groups (Zhang et al., 2022c). Therefore, effective management and conservation of alpine meadows should focus on nutrient accumulation in surface soil and promote root development to support healthier plant growth.

### 4.3 Allocation patterns of ecosystem carbon storage among grazing patterns

Vegetation C storage in the alpine meadow ecosystem of the Tibetan Plateau has been relatively understudied, with notable differences observed in the results across various studies. Specifically, Ma et al. (2010) reported a vegetation C storage of 337.6 g·m^-^² in the studied area, while a study by Fan et al. (2008) found a significantly higher value of 2170 g·m^-^². This discrepancy may primarily arise from two factors: biomass and biomass C concentration. Currently, biomass data collected from field samples are quite limited, particularly for root biomass, which is more challenging to obtain than aboveground biomass (Gao et al., 2008). Previous research has often used the ratio of aboveground to belowground biomass to estimate root biomass (Piao et al., 2007). However, seasonal changes in root biomass and varying ratios under different grazing patterns create uncertainties in this method. In this study, root C storage was calculated more accurately by directly measuring all biomass and assessing the C concentration (Cao and Chen, 2017; Johnstone, 2021).

The alpine meadows of the Tibetan Plateau play a crucial role in China’s grassland ecosystem, comprising about 54.5% of the C pool (Luan et al., 2014). Compared to the CG pattern, both BG and RG patterns showed significantly higher C storage in aboveground biomass, roots, litter, and total vegetation, aligning with the findings of Li et al. (2017). This was mainly attributed to the lack of direct vegetation damage from livestock grazing under the BG and RG patterns. Additionally, moderate grazing enhanced the growth of perennial herbs and promoted dense root systems, further contributing to C storage in alpine meadow vegetation (Li et al., 2017). Ecosystem C pools can be divided into two main components: vegetation and soil. The dynamics of both components significantly influence ecosystem structure and function (Li et al., 2024). The study found that moderate grazing, particularly under BG and RG patterns, significantly enhanced C storage in alpine meadow ecosystems. Moreover, C storage in alpine meadows was notably higher than the average C storage of Chinese grassland ecosystems, measured at 8820.5 g·m² (Fang et al., 2010). Alpine meadows exhibited significantly higher ecosystem C storage compared to the temperate steppe (8313.45 g·m²) and temperate desert (4905.36 g·m²) on the Loess Plateau (Li et al., 2024). These differences may arise from geographical factors such as hydrothermal conditions, ecological characteristics, grassland species composition, and soil types (Chen et al., 2023). The study revealed that alpine meadow ecosystems had higher C storage compared to previous research by Li et al. (2017). This discrepancy may result from variations in grazing years and differences in calculation methods. Our study used the equivalent mass method to estimate soil C storage, while Li et al. (2017) calculated it using concentration, soil bulk density, and soil thickness, without accounting for soil texture. Despite these methodological differences, our results align with established trends in C storage under various grazing patterns (Li et al., 2017). Furthermore, our study showed that belowground biomass in each functional group decreased from BG to RG, resulting in a corresponding decline in root C storage, consistent with findings by Gao et al. (2008). These findings enhance our understanding of the effects of grazing patterns on C cycling in alpine meadows.

### 4.4 Drivers of ecosystem C storage

The structural equation modeling analysis indicated that grazing patterns indirectly affect ecosystem C storage by altering the C: N: P stoichiometry and biomass allocation of vegetation. This highlights the direct response of vegetation’s C: N: P balance and biomass structure to grazing in the Tibetan Plateau alpine meadows. Furthermore, the study identified grasses as a key functional group influencing ecosystem C storage due to their efficient photosynthetic C fixation and rapid biomass accumulation. AGB of grasses showed a significant positive correlation with ecosystem C storage, with a standardized path coefficient of 0.78. This observation matches the dominance of grass species under grazing, as their large AGB directly boosts ecosystem C storage (Lan et al., 2023). In contrast, while legumes grow more slowly, their ability to fix nitrogen boosts N levels. This helps other plants grow, which in turn supports ecosystem C storage (Kokkini et al., 2025). Therefore, when developing strategies for managing ecosystem C storage, it is important to consider the biomass traits of different plant functional groups and how they adapt to environmental conditions.

Further analysis showed that vegetation C: N: P stoichiometry influences ecosystem C storage through two pathways: directly impacting C storage and indirectly optimizing biomass allocation among functional groups. For example, in the BG pattern, lower N: P ratios in the aboveground parts of grasses influence N cycling by slowing N mineralization, which helps accumulate soil organic matter. At the same time, this enhances ecosystem C storage through significant increases in grass biomass (Su and Xu, 2021; Wang et al., 2022a). Conversely, the CG pattern reduces the C: N ratio in legumes, which activates their symbiotic N fixation. However, in the BG pattern, overgrazing significantly reduces ecosystem C storage compared to the CG pattern. These mechanisms suggest that vegetation C: N: P stoichiometry affects ecosystem C storage by influencing biomass indirectly. This highlights the important role of the “stoichiometric steady state–biomass balance” coupling mechanism in grassland C management.

In summary, under grazing disturbances, the biomass and stoichiometry of different plant functional groups work together to affect ecosystem C storage in alpine meadows. To maximize C sequestration, effective grazing management should balance the biomass of grasses and legumes. This creates a positive feedback loop of “increased productivity—activated soil nitrogen,” which enhances ecosystem C storage (Liu et al., 2017; Wang et al., 2022a). Additionally, applying moderate disturbance strategies is essential to prevent soil C loss from overgrazing. By using integrated management approaches that involve “grazing patterns—stoichiometry regulation—biomass management,” the C sink potential of alpine meadows can be greatly improved. This offers sustainable ecological strategies for grasslands that can help mitigate global climate change.

## 5 Conclusion

In conclusion, this study demonstrates that grazing patterns significantly influence vegetation C: N: P stoichiometry and C storage in alpine meadows. The BG pattern ecosystem C storage was the highest, whereas the CG pattern led to a notable decrease in C levels. Additionally, the RG pattern enhanced nutrient enrichment in grasses. Grazing patterns indirectly affect ecosystem C storage by altering C: N: P stoichiometry, notably with increased aboveground N: P ratios in grasses and decreased C: N ratios in sedges and legumes under the CG pattern. The AGB of grasses was found to be a critical driver of ecosystem C storage, showing a strong positive correlation (*R*² = 0.96) with C variations. Therefore, maintaining a balanced biomass of functional groups and optimal C: N: P stoichiometry is essential for preserving ecosystem C storage in the context of grazing disturbances.

## Data Availability

The raw data supporting the conclusions of this article will be made available by the authors, without undue reservation.

## Supplementary material

Supplementary Figure 1 Geographical context of the study area.

Supplementary Figure 2 Relationship of carbon stocks with vegetation, root biomass **(a)** and C: N: P stoichiometry **(b, c)**. **P* < 0.05; ***P* < 0.01; ****P* < 0.001.
