# Supplementary material for: Vegetation C: N: P stoichiometry and ecosystem carbon storage under different grazing patterns on the Tibetan Plateau
Source: Front Plant Sci. 2025 Nov 26;16:1651605. doi: 10.3389/fpls.2025.1651605 (PMC12689571; doi:10.3389/fpls.2025.1651605)
Supplement: Supplementary Figure 1 — Geographical context of the study area. [file SupplementaryFile1.docx]

**Supplementary materials**

**Vegetation C: N: P stoichiometry and ecosystem carbon storage under different grazing patterns on the Tibetan Plateau**

Guoxing He ^1,2^, Xiaoni Liu ^1,2*^, Yali Li ^1,2^ and Tong Ji ^1,2^

1 Key Laboratory of Grassland Ecosystem, Ministry of Education, Pratacultural College, Gansu Agricultural University, Lanzhou, 730070, Gansu, China;

2 Sino-U.S. Center for Grazing Land Ecosystem Sustainability, Lanzhou, 730070, Gansu, China

*CORRESPONDENCE: Xiaoni Liu. E-mail: [liuxn@gsau.edu.cn](mailto:liuxn@gsau.edu.cn)


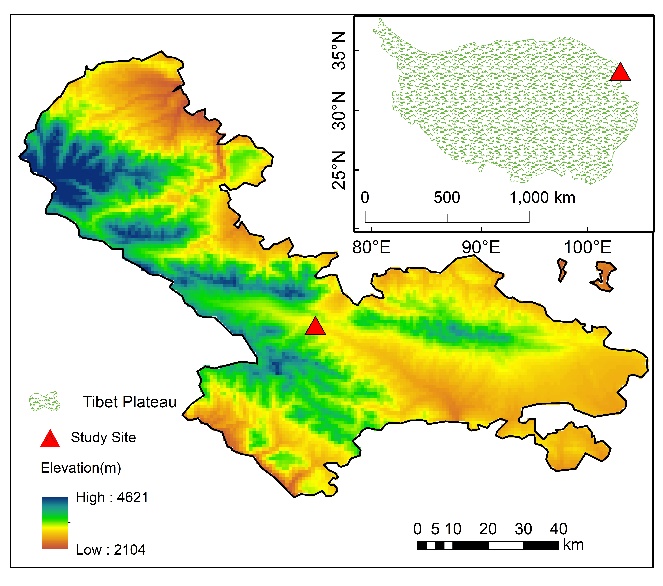


FIGURE S1 Geographical context of the study area.


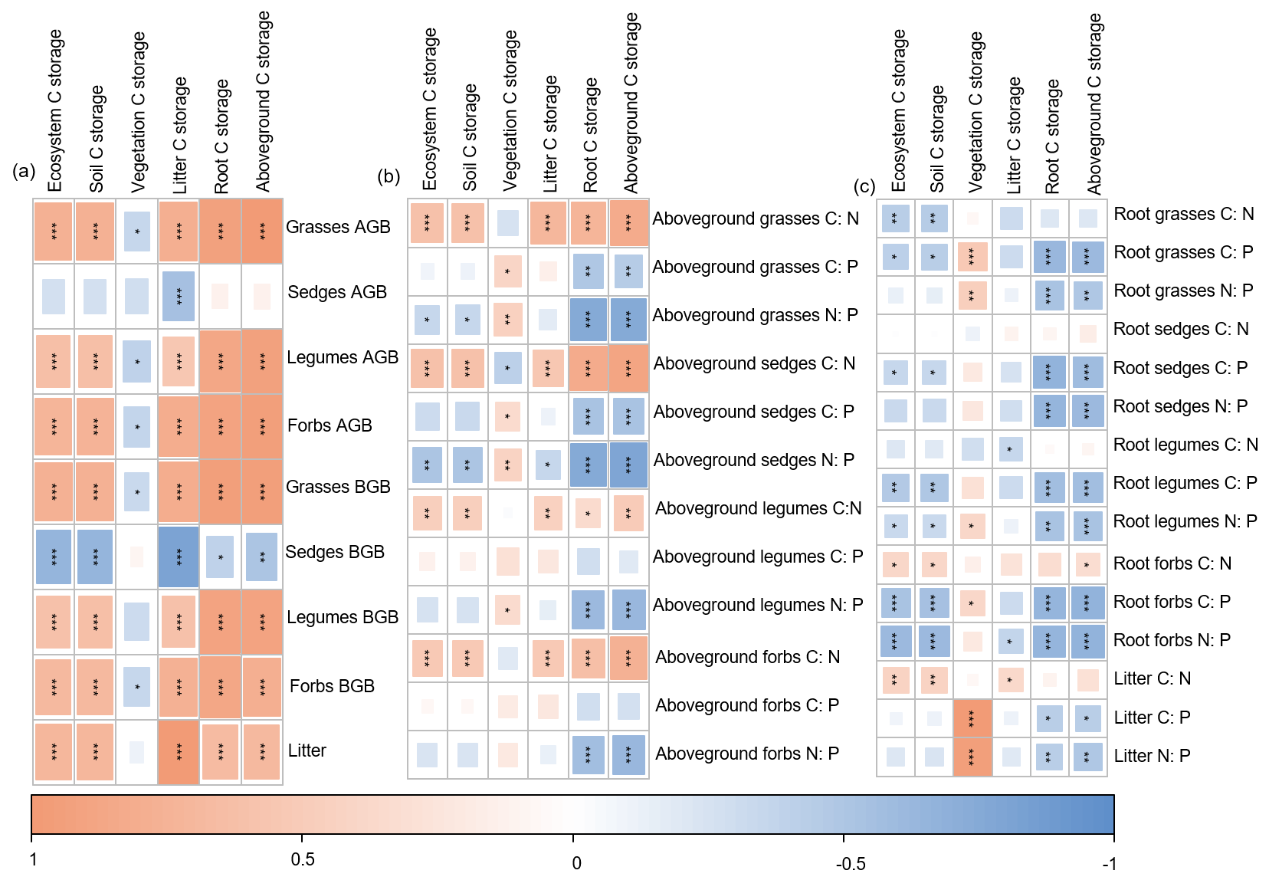


FIGURE S2 Relationship of carbon stocks with vegetation, root biomass (a) and C: N: P stoichiometry (b and c). **P* < 0.05; ***P* < 0.01; ****P* < 0.001.
